# Stability of Conducting Polymer-Coated Carbon Microfibers for Long-Term Electrical Stimulation of Injured Neural Tissue

**DOI:** 10.3390/polym16142093

**Published:** 2024-07-22

**Authors:** Hugo Vara, Gabriel Raúl Hernández-Labrado, Alexandra Alves-Sampaio, Jorge E. Collazos-Castro

**Affiliations:** 1 Neural Repair and Biomaterials Laboratory, Hospital Nacional de Parapléjicos (SESCAM), Finca la Peraleda S-N, 45071 Toledo, Spain; hvara@sescam.jccm.es (H.V.); aalves@sescam.jccm.es (A.A.-S.); 2 Escuela de Ingeniería Industrial y Aeroespacial, Universidad de Castilla-La Mancha, Avda. Carlos III, 45071 Toledo, Spain; gabrielr.hernandez@uclm.es

**Keywords:** carbon microfiber, conducting polymer, PEDOT, electrode, neural, electrical stimulation, stability, spinal cord injury

## Abstract

Electroactive microfiber-based scaffolds aid neural tissue repair. Carbon microfibers (CMFs) coated with the conducting polymer poly(3,4-ethylenedioxythiophene) doped with poly[(4-styrenesulfonic acid)-*co*-(maleic acid)] (PEDOT:PSS-*co*-MA) provide efficient support and guidance to regrowing axons across spinal cord lesions in rodents and pigs. We investigated the electrical and structural performance of PEDOT:PSS-*co*-MA-coated carbon MFs (PCMFs) for long-term, biphasic electrical stimulation (ES). Chronopotentiometry and electrochemical impedance spectroscopy (EIS) allowed the characterization of charge transfer in PCMFs during ES in vitro, and morphological changes were assessed by scanning electron microscopy (SEM). PCMFs that were 4 mm long withstood two-million-biphasic pulses without reaching cytotoxic voltages, with a 6 mm length producing optimal results. Although EIS and SEM unveiled some polymer deterioration in the 6 mm PCMFs, no significant changes in voltage excursions appeared. For the preliminary testing of the electrical performance of PCMFs in vivo, we used 12 mm long, 20-microfiber assemblies interconnected by metallic microwires. PCMFs-assemblies were implanted in two spinal cord-injured pigs and submitted to ES for 10 days. A cobalt–alloy interconnected assembly showed safe voltages for about 1.5 million-pulses and was electrically functional at 1-month post-implantation, suggesting its suitability for sub-chronic ES, as likely required for spinal cord repair. However, improving polymer adhesion to the carbon substrate is still needed to use PCMFs for prolonged ES.

## 1. Introduction

Besides triggering neuronal activity, applied electrical stimulation (ES) can modulate diverse cell behaviors including proliferation, migration, elongation of cytoplasmic processes, and differentiation [1,2,3,4]. This makes it possible to use ES to promote wound healing and organ regeneration [5]. Nevertheless, administering ES by electrodes external to the targeted organs may not be sufficient to induce significant tissue repair. For instance, in the specific case of traumatic central nervous system (CNS) damage, the dura matter attenuates the electric current supplied by epidural electrodes, compromising its usefulness to induce neural regeneration [6]. Consequently, penetrating neural electrodes may be more efficient to achieve this goal. On the other hand, cavities devoid of cell-adhesive molecules and guidance cues form when dead cells are removed after CNS injury [7,8], raising the additional need to provide an appropriate substrate for neural regrowth. Therefore, ES must be integrated with complementary approaches to tackle the multiple hurdles to brain and spinal cord repair. Implantable, biofunctionalized electroactive tissue scaffolds may solve those challenges by providing a substrate bearing physical and biochemical cues to support neural regeneration, together with dynamical ES patterns to potentiate functional tissue repair [9,10,11].

The superior electrical, mechanical, and non-immunogenic features of carbon microfibers (CMFs) [12,13,14,15] confer them great potential for the fabrication of durable neural recording electrodes, which apply no electric current to the biological tissues. However, the charge injection capacity of CMFs must be improved for their use as active electrodes in neural stimulation. The requirements of electric charge injection to trigger neuronal activity are relatively high [16,17]. CMFs have a low electric capacitance (about 0.01 mF/cm^2^ [18]) and therefore produce large voltage excursions when pulsed with neural-activating pulsed current [19], which may damage the microfibers themselves and/or the surrounding cellular elements. Coating CMFs with conducting polymers such as poly(3,4-ethylenedioxythiophene) (PEDOT) increases their electric capacitance by 2–3 orders of magnitude (to about 3 mF/cm^2^ for a 0.5–1 μm thick PEDOT layer [18]), and thus reduces the microfiber potential to electrochemically safe values for electric pulse intensities above neural activation thresholds [19].

PEDOT:PSS-*co*-MA-coated CMFs (PCMFs) of diameter between 6 and 10 μm are especially suitable for CNS repair because they can be surgically handled and implanted parallel to the axons, thus minimizing additional tissue damage while bridging injury sites [9,20]. Moreover, PCMFs can be of any chosen length, as needed for human neurological lesions that range from a few mm to several cm [21,22]. When appropriately modified with biomolecules, PCMFs support long-distance, guided axonal growth either in vitro [18,23] or in vivo [20,24].

Long-term in vivo studies on neural repair [20,24] have used bundles of loose (i.e., non-interconnected) PCMFs because of the difficulty of producing electrically interconnected microfiber scaffolds suitable for chronic implantation. This has precluded applying ES through the implanted MFs to promote neural regeneration in vivo. Nevertheless, the electrical performance of short (50–250 μm) PCMFs has been investigated for neuroscience applications in simpler in vitro or acute in vivo experiments, showing that they can be advantageously used for the ultrasensitive recordings of neural cell activity/spikes [25] or spinal cord stimulation [19]. In the latter study, microelectrodes made of 7 μm diameter CMFs coated with PEDOT:PSS-*co*-MA were used to apply current-controlled biphasic electric pulses at the spinal cord of anesthetized rats. Electrodes that are 250 μm long were effective in activating specific spinal motoneurons at low stimulus thresholds (from 28 μA to 46 μA in the cathodic phase) and electrochemically safe voltages (between −0.9 V and +0.4 V). Intriguingly, for the same applied current, stimulation with PCMFs produced higher muscle electrical activity compared to stimulation using uncoated CMFs. However, despite the superior performance as neural stimulating electrodes, PCMFs deteriorated after a few thousands of stimulation pulses, producing unsafe, large voltage excursions (>1.5 V) during electric current application.

Two complementary strategies can be used to increase the stability of electroactive PCMFs, namely reducing the charge density applied per pulse, or improving PEDOT adhesion to the CMF surface. Because human CNS injuries are large, ranging from some mm to several cm in length [21,22], here we explore the first of those strategies reducing charge density by means of increasing the microfiber length to the order of mm. We investigate the minimal length requirements of PCMFs to withstand long-term ES with electrochemically safe voltage excursions in vitro. Because information about the optimal ES parameters to promote CNS regeneration through an electroactive scaffold is still unavailable, for the present study, we selected an ES protocol of about 1.6 million pulses known to increase the sprouting of uninjured corticospinal axons [3]. We administered pulses of 50 μA/200 μs/phase for the assessment of individual PCMFs in vitro, and 200 μA/200 μs/phase for the preliminary studies of PCMFs assemblies in vivo, because those parameters are sufficient for the activation of spinal cord neurons [19] or axonal tracts [26], respectively. Moreover, we identify key EIS parameters that may detect the deterioration of the MFs before a significant increase in voltage excursions, and present a preliminary proof of concept that interconnected PCMF assemblies may be useful for the safe, sub-chronic ES of the injured porcine spinal cord.

## 2. Materials and Methods

### 2.1. Preparation of Individual PCMFs

Individual 7 μm diameter CMFs (Goodfellow, Huntingdon, UK) were introduced into borosilicate capillaries (A-M Systems, Sequim, WA, USA) by applying a negative pressure. A side of the capillary was sealed with silicone, allowing the CMFs to protrude from it. Subsequently, the microfibers were cut to a length of 1, 2, 4, or 6 mm for electrochemical testing. CMF lengths were chosen in anticipation of the eventual in vivo applications related to cavities produced by spinal cord injury models in rats and pigs, and also based in preliminary data in vitro spanning a range from worst to best electrochemical performance and then setting intermediate length points. At the rear end of the capillary, the CMFs were trimmed and covered with colloidal graphite (Agar Scientific, Stansted, UK), which was also applied to the internal and external surfaces of the glass. Finally, a strip of conductive adhesive tape was stuck on the dried graphite to allow a mechanically stable electric connection.

PEDOT doped with PSS-*co*-MA was electropolymerized on CMFs by galvanostatic method at room temperature, using an Autolab PGSTAT302 potentiostat/galvanostat (Eco Chemie, Utrecht, The Netherlands) in three-electrode cell configuration. A platinum foil served as counter-electrode (CE), and an Ag/AgCl electrode (MI-402, Microelectrodes, Inc., Bedford, NH, USA) was the reference electrode (RE). The electropolymerization solution contained NaCl-free phosphate buffer (PB), 15 mM 3,4 ethylenedioxythiophene (Sigma-Aldrich, St. Louis, MO, USA), and 20 mM PSS-*co*-MA (Sigma-Aldrich). A constant anodic current of 1 μA/mm^2^ of CMF gross surface area (GSA) was applied through the microfiber for 1920 s. The values of GSA, electric current intensity, and resulting polymerization charge for the tested CMF electrode lengths are shown in Table 1.

### 2.2. Electrochemical Characterization of PCMFs

The electrical performance of PCMFs was assessed in a three-electrode configuration within a custom-made miniature electrochemical cell (hereafter referred to as the “minicell”) designed to reduce the volume of the electrolyte and the dispersion of the electric current. Artificial cerebrospinal fluid (ACSF) was used as the electrolyte with the following composition (in mM): NaCl, 120; KCl, 2.5; NaH_2_PO_4_, 1.0; MgCl_2_, 1.2; CaCl_2_, 2.5; NaHCO_3_, 26.2; glucose, 11; pH 7.4 when equilibrated with 95% O_2_/5% CO_2_. The minicell consisted of a hollow cylinder (22 mm long, 2.5 mm inner diameter, 3.5 mm outer diameter), with a 2 mm diameter MI-402 Ag/AgCl RE placed in one of the two edges. A platinum wire (0.127 μm diameter), coiled around the base of the RE shaft (2 mm diameter), served as CE. This edge was watertight sealed with silicone. Two lateral tubes were connected to the main cylinder close to the RE side to allow for the refilling of ACSF when necessary. The PCMFs to be tested were individually introduced through the opposite edge, with the tip of the microfiber separated from the RE by 2 mm. In these conditions, a train of three biphasic pulses was administered at a frequency of 300 Hz through the PCMF while the voltage was monitored at 1 μs resolution. The electric pulses were symmetric biphasic, cathodic-first rectangular waves, of 200 μs duration and 50 μA amplitude per phase. A 20 μs interphase at zero current was introduced between the cathodic and anodic phases for a more precise determination of the maximum cathodic and anodic polarization potentials (E_mc_ and E_ma_, respectively), as proposed by Cogan [17]. E_mc_ and E_ma_ were measured immediately after the end of the cathodic and anodic phases, when the current intensity was zero (i = 0).

The electrical behavior of PCMFs was also studied by electrochemical impedance spectroscopy (EIS), using the minicell with the same ACSF electrolyte and electrode configuration. For this, 20 mV sine waves were applied at 26 logarithmically spaced frequencies from 10^5^ to 1 Hz through the PCMFs. An equivalent electrical circuit contained three elements in series as proposed by Bobacka et al. [27], considering generalized components as suggested by Collazos-Castro et al. [4], allowed to fit EIS data in the frequency range between 1 Hz and 1 kHz (Section 3.1). For this, a code was implemented in MATLAB^®^ (version R2020a) using an optimization method based on nonlinear least-squares.

### 2.3. Assessment of PCMFs Stability In Vitro

The 25 Hz biphasic, cathodic-first rectangular waves of 200 μs and 50 μA per phase were applied through the PCMFs at 37 °C in the minicell. As explained above, we searched for microfiber lengths that were able to stand at least 1.6 million pulses without producing unsafe voltage excursions. However, for the shortest (1 mm) microfibers, the stimulation was halted after 800k pulses because of their premature deterioration (see results). PCMFs of other lengths received 2 million pulses. In every case, ES was paused each 200k pulses to assess the performance of the microfibers by high-resolution (1 μs) chronopotentiometry. The EIS measurements were conducted every 400k pulses. After completing the ES protocol, the PCMFs were imaged by scanning electron microscopy (SEM) at 2 kV, using a FEI Verios 460 microscope in high vacuum mode and a “through the lens” detector. This allowed correlating the electrical behavior of the microfibers with their morphological changes.

### 2.4. Fabrication of PCMFs Assemblies

Implantable assemblies of 12 mm long, 20 parallel microfibers, separated 500 μm from each other, were fabricated for a preliminary assessment of PCMFs usefulness to apply ES in the damaged porcine spinal cord. In brief, CMFs were interconnected at the middle of their lengths through metallic (35N LT cobalt-base alloy, or stainless steel) microwires (12.7 and 12.5 μm in diameter, respectively) by Axon’ Cable SAS (Montmirail, France). The wires were glued to the CMFs with medical grade electroconductive epoxy (H20E, Epoxy Technology, Billerica, MA, USA), and the wires and the microfiber/wire junctions were coated with electrically insulating silicone. The cobalt alloy and stainless steel microwires of the PCMFs assemblies ended in a nano-D connector (Axon’ Cable SAS, Montmirail, France) to enable electrical interfacing. The assembled CMFs were then coated with PEDOT:PSS-*co*-MA using the electrochemical parameters described in Section 2.1. After washing and drying the assemblies, they were sterilized with paraformaldehyde gas and functionalized for cell attachment with a multilayer of poly-L-Lysine, heparin, basic fibroblast growth factor, and fibronectin [23]. Finally, the assemblies were embedded in a fibrin gel as previously described for the implantation of loose PCMFs in pigs with contusion spinal cord injury (SCI) [24]. For the current case, the fibrin gel was formed with the shape and volume of a porcine C6 cervical spinal cord hemi-segment to fill the neural tissue defects as described below.

### 2.5. In Vivo Testing of PCMFs-Assemblies

Cervical hemisection SCI with exeresis of 1 cm of the C6 spinal cord segment was produced in 2-month-old domestic pigs (*Sus scrofa domesticus*) under inhalational anesthesia, as described by Cerro et al. [28]. The fibrin gel-coated PCMF assemblies were implanted in the resulting spinal cord cavity. Durorrhaphy was performed to keep the implant in place while allowing the metallic microcables to exit the meningeal ensheathment. The assembly’s microcables were connected to a transcutaneous extensible cable, which was interfaced to a potentiostat/galvanostat (Biologic VSP20) for applying current-controlled electric pulses and simultaneously measuring the PCMFs voltage transients. For this, two epidurally placed 316 L stainless steel (SS) disks (3 mm in diameter, 50 μm thickness) were used as counter-electrode and pseudo-reference-electrode. The metal was exposed on the side facing the spinal cord, and the other side was insulated with silicone. In both pigs, biphasic rectangular pulses of −200/+200 μA (30 μA/mm^2^), 200 μs/phase, 45 ms at 300 Hz, repeated each 2 s, 6 h/day, for 10 days, were applied through the assemblies. ES was initiated at 72 h after the SCI and implant surgery. During the 6 h of daily ES, the pigs rested on a soft mattress and wore a harness that restricted their movements. Nevertheless, the animals could feed and drink ad libitum and had no visible discomfort associated with the electric pulses. To assess the electrical function of the assemblies, voltage transients were recorded in vivo during the application of test trains consisting of 10 biphasic electric current pulses (as before, but with an added i = 0 current interphase with a duration of 40 μs), at 300 Hz, using an acquisition frequency of 50 kHz. This test was initially performed a few minutes after the SCI and assembly implantation surgery, and was repeated daily, before and after each of the stimulation sessions.

## 3. Results

### 3.1. General Properties of PCMFs

**Figure 1:**
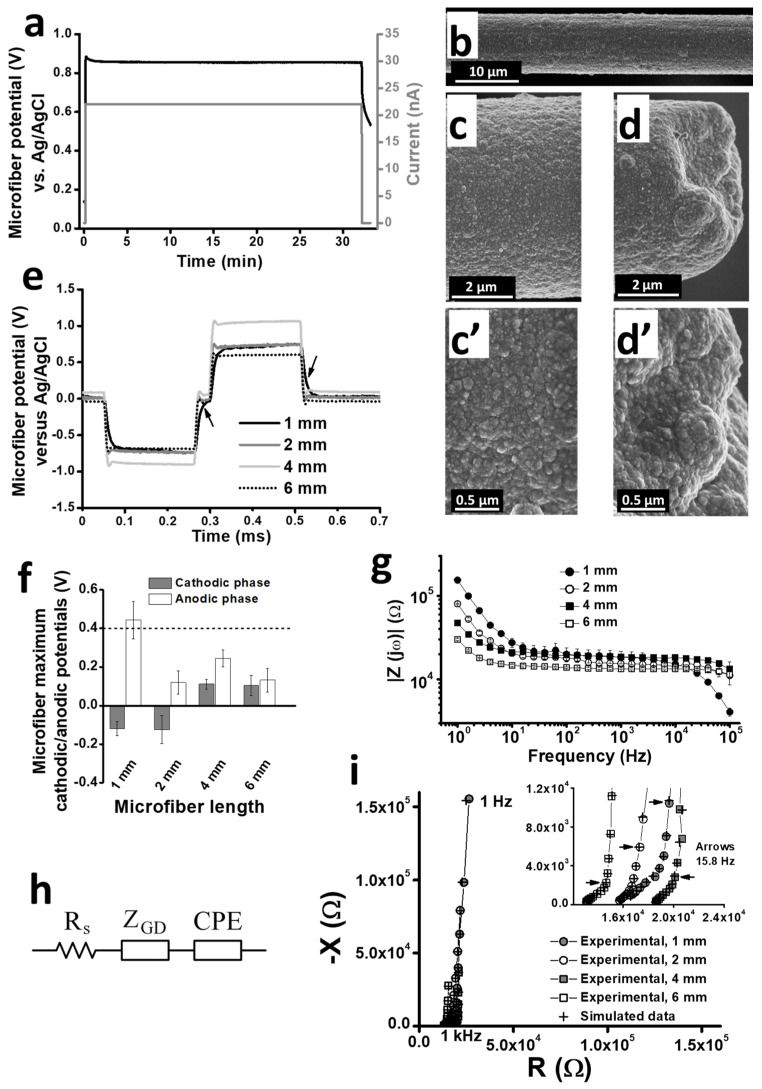
Characterization of PCMFs. (**a**) Illustration of the typical polymerization potential for PEDOT:PSS-*co*-MA when applying constant current through 1 mm CMFs. (**b**) SEM image of the PCMF shaft after the polymerization. (**c**,**c’**) High-resolution SEM images of the microfiber shaft, showing the rough, nanoporous structure of the polymer. (**d**,**d’**) Same details of the polymer structure at the microfiber tip. (**e**) Representative traces of voltage transients produced by the application of −50/+50 μA biphasic rectangular pulses through the PCMFs of different lengths. Arrows indicate the E_mc_ and E_ma_ measured at zero current. (**f**) Average values of E_mc_ and E_ma_ from the tested PCMFs: 1 mm (n = 3), 2 mm (n = 3), 4 mm (n = 2), 6 mm (n = 4). Dashed line indicates water electrolysis potential value. (**g**) EIS Bode plots from the same PCMFs. When not visible, error bars are smaller than symbols. (**h**) Equivalent electrical circuit proposed for impedance data fitting. The model consists of three elements in a series: R_s_, electrolyte resistance; Z_GD_, generalized Warburg diffusion impedance; CPE, constant phase element modeling the pseudo-capacitance C_d_. (**i**) Nyquist plot from a representative PCMF of each length, superimposing experimental and simulated data. The arrows signal impedance values for a frequency of 15.8 Hz.

PEDOT:PSS-*co*-MA polymerization voltage and morphology were similar irrespective of CMF length. During electropolymerization, the microfiber potential rose to a plateau of about 0.85 V that remained constant until current switch-off (Figure 1a). A continuous and homogeneous polymer layer was formed (Figure 1b). The characteristic nodular and highly porous nanostructure of the polymer [18,19] was evenly distributed along the shaft (Figure 1c,c’) and the tip (Figure 1d,d’) in all PCMFs.

The measurement of the maximum cathodic and anodic voltage excursions produced by the prepared PCMFs when pulsed at 50 μA (Figure 1e) showed that all microfiber lengths worked in safe values (i.e., between −0.9 V and +0.4 V), except for the 1 mm microfibers that had a somewhat higher anodic voltage (+0.44 ± 0.1 V, Figure 1f). As expected, the EIS analyses revealed an inverse relationship between microfiber length and impedance modulus, mainly at the lowest frequencies. Figure 1g shows the Bode plot for the averaged impedance of electrodes of each length. Significant differences (*p* < 0.05, ANOVA) existed among all length groups at all frequencies lower than 10 Hz, and up to a frequency of 15.8 Hz between 1 and 6 mm long electrodes.

Figure 1h shows the electrical circuit proposed for fitting EIS data, depicting a resistance (R_s_) that represents the electrolyte resistance, a constant phase element (CPE) modeling the almost ideal capacitance (C_d_) of the conducting polymer, and a generalized finite-length Warburg diffusion impedance (Z_GD_) representing ion diffusion-dependent electric charge transport in the polymer. Equations (1) and (2) are mathematical expressions for CPE and Z_GD_, respectively.

(1) $$CPE=\frac{1}{{\left(j\omega \right)}^{\alpha }C_{d}}$$

(2) $$Z_{GD}=\frac{R_{D}coth\left({\left(j\omega \tau_{D}\right)}^{\beta }\right)}{{\left(j\omega \tau_{D}\right)}^{\beta }}$$

Z_GD_ is determined by the diffusional time constant (τ_D_) and the diffusional resistance of the polymer (R_D_). The last two parameters for Z_GD_ can be related to obtain the diffusional pseudo-capacitance of the polymer coating (C_D_ = τ_D_/R_D_). Equivalent circuit fitting provided values for α higher than 0.9, indicating that the CPE is comparable to an ideal capacitor, whereas values for β were close to 0.5, thus allowing the identification of the generalized Warburg impedance with the ‘classical’ finite-length open Warburg element [29]. On the other hand, the Nyquist plots showed a ~45° diffusion line at high frequencies followed by a ~90° capacitive line at low frequencies (Figure 1i) for all microfiber lengths, which is equivalent to the response of Z_GD_ and represents ionic diffusion in the polymer. Introducing C_d_ in the model was necessary to account for the slight deviation from the ideal diffusion behavior [27], and this parameter is related to the electronic capacitance in the bulk of the polymer, as suggested for mixed conductors [30]. Finally, the total polymer bulk capacitance (C_tot_) was calculated as C_tot_ = (1/C_d_ + 1/C_D_)^−1^. Fitting parameters and derived values for EIS data are shown in Table 2 for all PCMF lengths.

Solution resistance (R_s_) was relatively high (>10 kΩ) because of the small dimensions of the electrochemical cell and the electrical properties of the solution. Diffusional resistance (R_D_) was higher in 1 mm PCMFs compared to other lengths, suggesting that increasing the area coated by polymer facilitates ionic diffusion inside the polymer bulk. Electronic capacitance (C_d_) and diffusional pseudo-capacitance (C_D_) are known to increase with polymerization charge, and therefore with conducting polymer volume [4,27]; accordingly, both parameters increased with microfiber length. Nevertheless, for PCMFs, C_d_ was an order of magnitude higher than C_D_, whereas the inverse proportion was reported for PEDOT coatings on the electrodes of other materials and dimensions [4,27]. Possible explanations for this difference are discussed in Section 4.

### 3.2. Electric Charge Transfer during Long-Term ES

**Figure 2:**
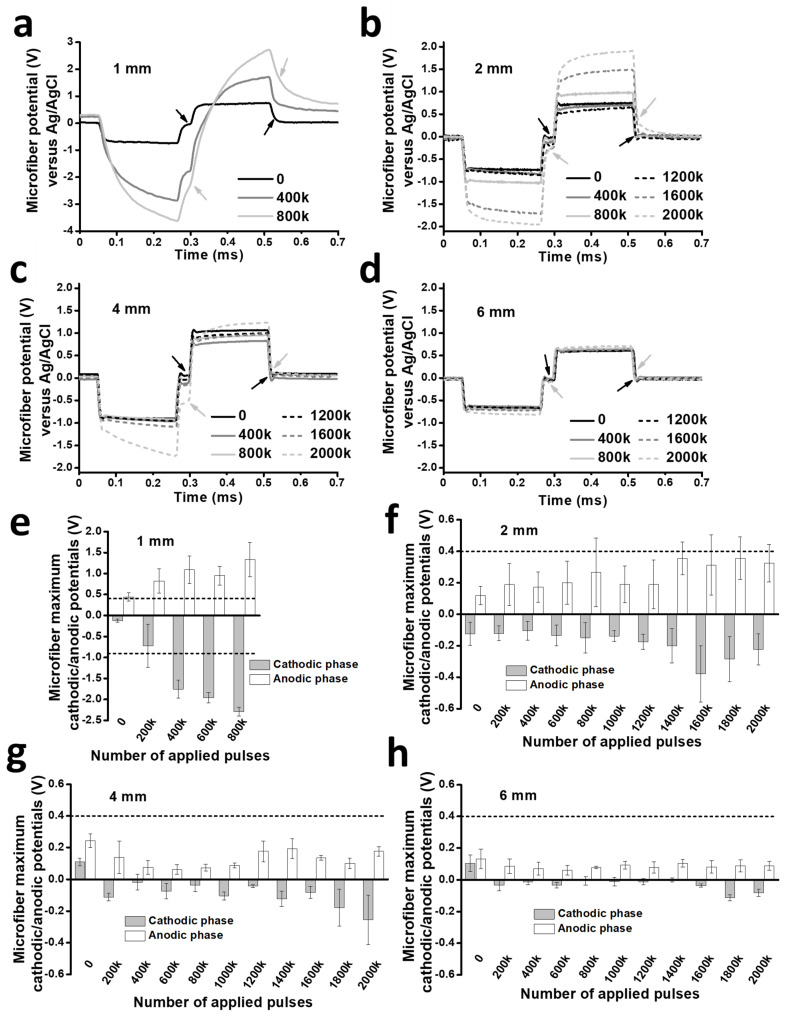
Evolution of PCMFs voltage transients as a function of the number of ES pulses. (**a**–**d**) Representative recordings of voltage transients from microfibers of different lengths. Arrows point to E_mc_ and E_ma_ potentials measured at zero current. (**e**–**h**) Average E_mc_ and E_ma_ values measured from all the tested PCMFs. Dashed lines indicate water electrolysis potential values.

Voltage transients measured as a function of the number of applied electric pulses are illustrated in Figure 2a–d for representative microfibers of each length, whereas the average maximum cathodic (E_mc_) and anodic (E_ma_) polarization from the different microfibers are plotted in Figure 2e–h. Because pulses of the same intensity (50 μA) were applied to all microfibers, the electric charge density applied per pulse progressively diminished from 36.52 μC/cm^2^/phase to 18.28 μC/cm^2^/phase, 9.14 μC/cm^2^/phase, and 6.09 μC/cm^2^/phase for microfibers of 1 mm, 2 mm, 4 mm, and 6 mm in length, respectively.

PCMFs that are 1 mm long deteriorated quickly. In this microfiber length, E_mc_ and E_ma_ increased at a fast rate with ES, with values of −0.72 ± 0.51 V and 0.81 ± 0.29 V, respectively, after 200k pulses (Figure 2a,e). By 400k, they reached −1.75 ± 0.21 V and 1.09 ± 0.33 V, respectively, clearly surpassing the safety levels. Because voltages continued to increase, ES was halted at 800k pulses.

The electrical performance of PCMFs improved with the microfiber length. On average, E_mc_ and E_ma_ were maintained within safe values in 2 mm long microfibers along the 2 million electric pulses (Figure 2b,f). However, when considered individually, some of the microfibers already exceeded safe Ema limits after 1800k–2000k pulses. On the other hand, all 4 mm long microfibers showed safe voltage excursions during the complete testing regime (Figure 2g), although some increase in the cathodic voltage started to appear from 1600k to 2000k pulses (Figure 2c,g). Finally, E_mc_ and E_ma_ were low and stable for the entire duration of the ES protocol for 6 mm long microfibers (Figure 2d,h). Even considering a slight increase in E_mc_ observed from 1600k pulses—similar to that of 4 mm electrodes—voltage remained very low and within the safe electrochemical window. Therefore, 6 mm appears to be the minimum length required for a PEDOT:PSS-*co*-MA-coated CMF to be able to sustain enduring, current-controlled (50 μA) ES.

### 3.3. EIS Follow-Up of Microfiber Electrical Performance

**Figure 3:**
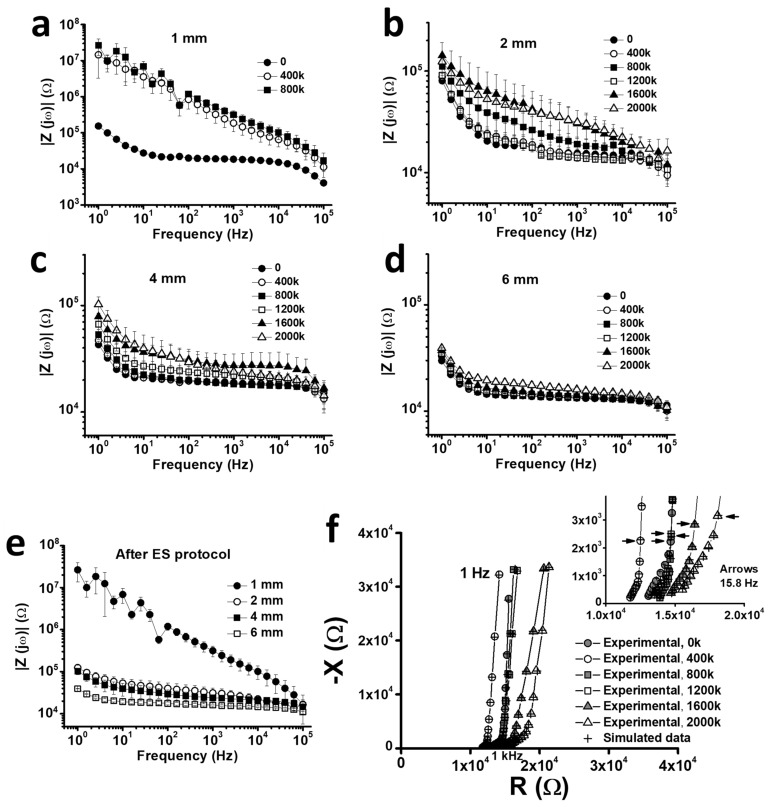
Changes in PCMFs impedance as a function of the number of ES pulses. (**a**–**d**) Bode plots of data averaged from all PCMFs of each length. (**e**) Bode plot of impedance data for all PCMF lengths at the end of the ES protocol, i.e., 2 million pulses for 2, 4, and 6 mm microfibers, and 800k pulses for 1 mm microfibers. (**f**) Nyquist plot from a 6 mm long PCMF along the whole ES protocol. R(Ω) and X(Ω) are the real and imaginary parts of the impedance, respectively. Arrows indicate impedance values for a frequency of 15.8 Hz.

EIS data agreed with the observations of voltage excursions and provided further insights into the stability of PCMFs as a function of microfiber length. EIS showed an early increase in the impedance modulus after 400k pulses in 1 mm long microfibers (Figure 3a). Such an increase reached two orders of magnitude in the lowest frequency ranges, for instance, from about 1.52 10^5^ Ω to 1.44 10^7^ Ω at 1 Hz. The Bode plot showed marked fluctuations in the impedance values for those microfibers at frequencies under 100 Hz. Those fluctuations appeared in all single microfibers and were likely due to their severe structural damage as visualized by SEM (Section 3.4). For 2 mm and 4 mm PCMFs, the impedance increase was limited to one order of magnitude along all frequencies and times of pulsing, with a higher progression at 1600k–2000k pulses (Figure 3b,c). In the case of 6 mm electrodes, changes were barely detected in the first 1600k pulses, and only a slight increase was apparent at 2000k pulses (Figure 3d). EIS values measured at the end of the ES protocol (2000k pulses for all electrodes except for 1 mm ones, which received 800k pulses) are illustrated for comparison of all microfiber lengths in Figure 3e.

EIS Nyquist plots for 6 mm microfibers at different ES periods showed the same profile as described in Section 3.1, i.e., a ~45° line at high frequencies consistent with a diffusion process, and a ~90° line at low frequencies representing capacitive behavior (Figure 3f). Despite the good electrical stability of those microfibers during the two-million pulses as indicated by the very low voltage excursions and impedance modulus in Bode plots, modelling EIS data (Table 3) revealed several changes in their electrical behavior that likely precede evident deterioration. For instance, the diffusional pseudo-capacitance (C_D_) lost about one-third of its initial value after the first pulsing periods, although later remained roughly constant. This caused a similar reduction in the total polymer bulk capacitance (C_tot_), which is dominated by C_D_. The electronic capacitance (C_d_) increased with the ES protocol, but it had little effect on the total capacitance. In turn, the diffusional resistance of the polymer (R_D_) lowered after 400k pulses, facilitating charge transfer, and subsequently increased, finally surpassing its initial value. Further increments in R_D_ and a reduction in C_tot_ will compromise the feasibility of using the microfibers for chronic ES.

### 3.4. SEM Correlates of PCMF Deterioration

**Figure 4:**
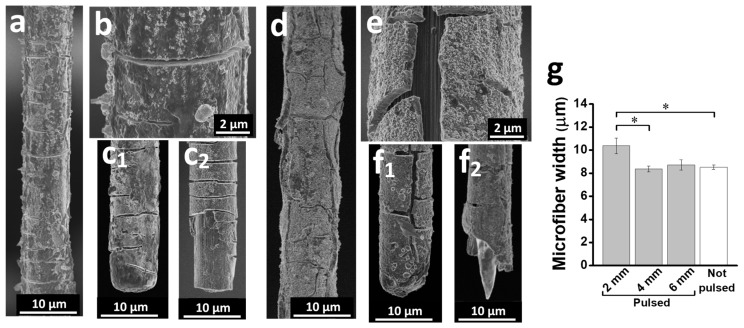
SEM imaging of PCMFs after ES. (**a**–**c**) The 6 mm PCMFs after 2 million electric current pulses, showing polymer surface degradation and cracking transversal to the microfiber longitudinal axis. Cracks appear both in the microfiber shafts (**a**,**b**) and tips. Examples of 6 mm PCMFs tips with different degrees of deterioration are shown in (**c_1_**,**c_2_**). The polymer of the tip shown in (**c_2_**) delaminated, but the carbon surface was intact. (**d**–**f**) SEM images of 1 mm PCMFs after 800k pulses, displaying extensive polymer cracking and wrinkling in the shafts (**d**,**e**) and damage in the tips (**f_1_**,**f_2_**), including the etching of the carbon microfiber itself (**f_2_**). (**g**) Evaluation of PCMFs width after 2 million electrical pulses compared to non-pulsed electrodes. * *p* (ANOVA) < 0.05.

The consequences of electrical pulsing on the structural integrity of the microfibers were studied by SEM. In contrast to non-pulsed microfibers (Figure 1b–d), electrically active microfibers showed numerous fissures in the polymer coating, exposing the underlying carbon surface. The 1 mm microfibers were more visibly damaged than 6 mm ones (Figure 4). In the 6 mm microfibers, the surface of the polymer had partially lost its original nanoporous structure, although a great part of the polymer was relatively well preserved, and the fissures were oriented mainly orthogonal to the microfiber axis (Figure 4a), whereas in 1 mm microfibers, the polymer was wrinkled and folded, with an increase in microfiber diameter, and fissures of varied shapes and orientations, including some longitudinal to the microfiber (Figure 4d). Higher magnification images also showed narrow (usually <1 μm wide) discontinuities in the polymer with apparently little exposition of the carbon surface in 6 mm PCMFs (Figure 4b), whereas wider polymer cracks with a larger carbon surface uncovering occurred in the shorter electrodes (Figure 4e). The tips of the microfibers showed different degrees of structural damage. Some of them preserved the polymer coating, with fissures as described for the shaft regions, whereas others lost the polymer, exposing tens of microns of the underlying carbon microfiber tip. Additionally, some tips of 1 mm electrodes had visible damage from the carbon microfiber itself, which acquired a pointed shape reminiscent of electrochemical etching [31].

The morphological damage of 2 mm and 4 mm long microfibers submitted to ES was somewhat intermediate between that of 1 mm and 6 mm microfibers. PCMFs’ diameter provided a simple quantitative indicator of the alterations in the polymer coating. As shown in Figure 4g, the diameter was not changed by 2 million pulses in 6 mm and 4 mm microfibers compared to non-pulsed controls, whereas a significant increase was observed in 2 mm ones. Because 1 mm electrodes received only 800k pulses, they were excluded from this analysis. Based on the SEM observations, we could exclude changes in the carbon microfiber core and attribute the increase in 2 mm PCMFs’ diameter to polymer swelling by solvated ions during ES in the aqueous electrolyte, and subsequent polymer cracking when ions were expelled or the PCMFs dried.

Considering electrochemical data and SEM imaging results together, a positive correlation existed between the loss of electrical performance and the structural damage of the microfibers after continued pulsing.

### 3.5. Long-Term ES through Implanted PCMFs Assemblies

A preliminary demonstration of the feasibility of using PCMFs for long-term ES in vivo was performed with assemblies of 20 parallel microfibers implanted in a model of porcine SCI. As described in the Section 2, 12 mm long, functionalized PCMFs-assemblies were embedded in a fibrin gel shaped to the size of the porcine cervical spinal cord cavity to facilitate both the handling of the assemblies and their retention at the lesion sites. Two assemblies were implanted in different pigs. The assemblies were identical except for the metallic microwire that interconnected the PCMFs, that was either a 35N LT cobalt-base alloy, or stainless steel (SS).

**Figure 5:**
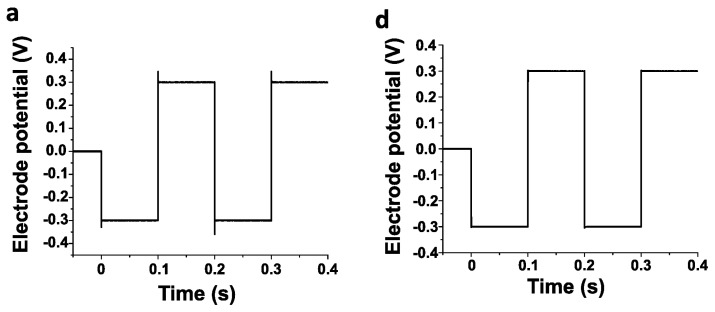

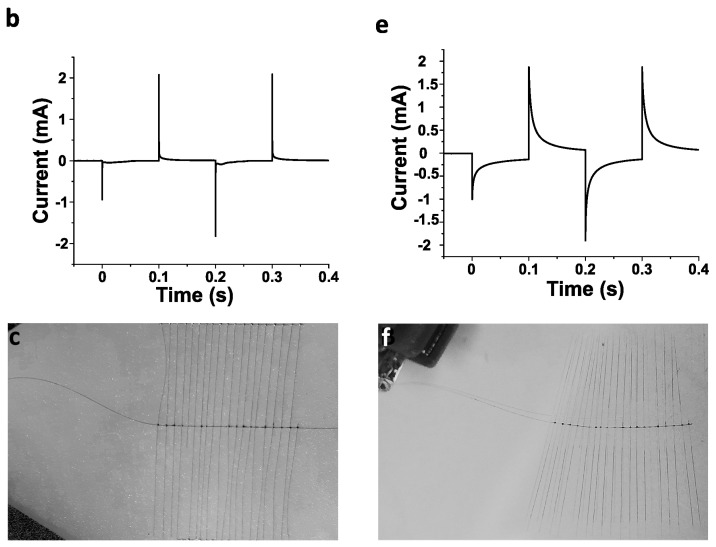
Effects of PEDOT:PSS-*co*-MA electrodeposition on a 35N LT-interconnected carbon microfiber assembly. (**a**,**d**) Voltage pulses (0.3 V biphasic rectangular waves, 0.1/s phase) applied for testing the assembly before (**a**) and after (**d**) forming the PEDOT coating. (**b**,**e**) Electric current responses of the carbon microfiber assembly when applying the voltage pulses before (**b**) and after (**e**) PEDOT electropolymerization. (**c**,**f**) Appearance of the assembly before (**b**) and after (**f**) the electrodeposition of the polymer.

Both types of assemblies could be successfully coated with PEDOT:PSS-*co*-MA. The appearance of the assemblies and their electric behavior before and after applying the polymer coating are illustrated in Figure 5. As previously reported for individual carbon microfibers and PCMFs [18], the PEDOT coating of the microfiber assemblies substantially increased the electric charge transfer. Voltage pulses (0.3 V) produced fast-decaying (μs) electric currents in the uncoated assemblies (Figure 5b), whereas enduring (100 ms) currents were detected after applying the PEDOT coating (Figure 5e).

**Figure 6:**
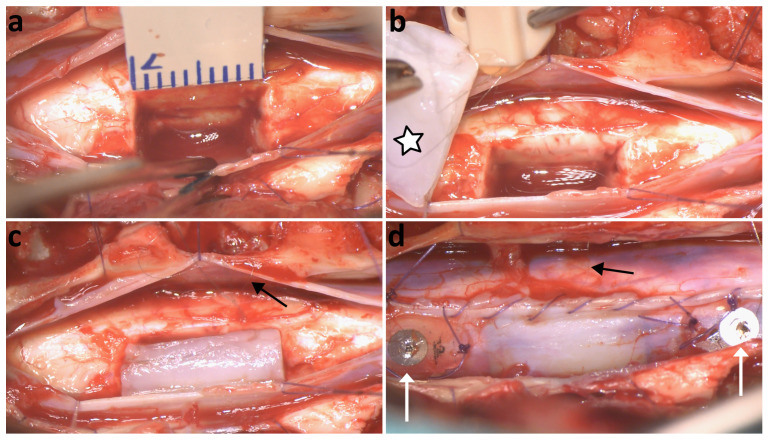
Porcine spinal cord injury model and implantation of a PCMF assembly. (**a**) Exeresis of the right side of the spinal cord segment C6 creating a 1 cm long cavity in the tissue. (**b**) Simultaneous handling of the fibrin gel (star) containing the interconnected assembly, together with the connector receiving the metallic microcable from the assembly. (**c**) Fibrin gel/PCMFs-assembly implanted into the lesion. (**d**) Meningeal suture and placement of epidural stainless-steel disks (white arrows) used as reference- and counter-electrodes at 1 cm from the lesion borders. Black arrows in (**c**,**d**) signal the metallic microcable exiting from the gel and the dura mater.

Subsequently to polymer electrodeposition, the assemblies could be conveniently sterilized, modified with biomolecules, introduced in the fibrin gel, and implanted into the injured porcine spinal cord, as illustrated in Figure 6.

**Figure 7:**
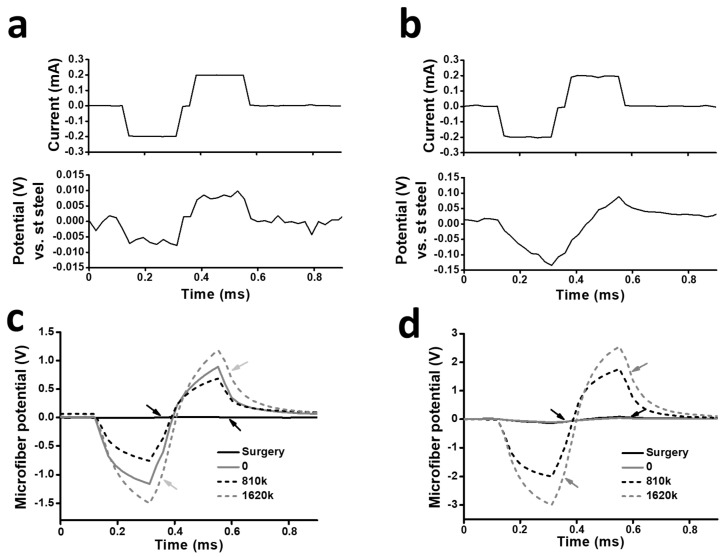

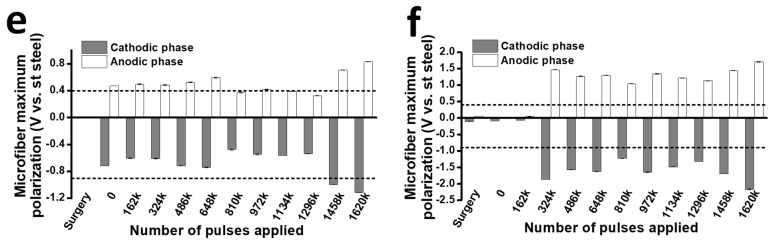
Electrical performance of PCMF assemblies implanted in the injured porcine spinal cord. (**a**,**c**,**e**) Data from the assembly interconnected by a 35N LT-microwire. (**b**,**d**,**f**) Data from the assembly interconnected by a stainless steel microwire. (**a**,**b**) Waveforms of the electric current applied by the potentiostat/galvanostat through the assemblies and the resulting voltage immediately after the implantation in the porcine spinal cord. (**c**,**d**) Voltage transients produced by the assemblies at the indicated number of pulses. Note that the vertical scales are different for (**c**,**d**). Arrows point to the maximum cathodic and anodic microfiber polarization (E_mc_ and E_ma_, respectively) measured after the cathodic or anodic phases when the intensity of the electric current i = 0. (**e**,**f**) Evolution of E_mc_ and E_ma_ for the assemblies in relation with the accumulated number of pulses. Data are expressed as mean ± standard error from 3 consecutive pulses of a chronopotentiometric test. Dashed lines indicate water electrolysis potential values.

Chronopotentiometry confirmed the correct electrical performance of the implanted devices immediately after implantation (Figure 7). Just after suturing the skin, both devices correctly applied biphasic electric pulses (−200/+200 μA, 40 μA/mm^2^) while producing safe voltage excursions, lower than 10 mV for the 35N LT-assembly and 140 mV for the SS-assembly (Figure 7a,b). After a 3-day post-operatory period, still without applying repetitive ES, voltage excursions were stable in the SS-assembly but increased to about −0.7 V/+0.5 V in the 35N-LT-assembly. Nevertheless, after starting ES, the voltages were relatively stable or even lowered in the 35N LT-assembly up to approximately 1.4 million pulses (about two weeks post-implantation), before increasing again and finally reaching the water electrolysis potentials (Figure 7c,e). The 35N LT-assembly was still electrically functional at the time of animal death (30 days post-implantation), although the voltages had further increased to −1.39 V/+1.04 V. In contrast, the SS-assembly deteriorated very soon, producing very large (−1.9 V/+1.5 V), unsafe potentials, at about 0.3 million pulses, one day after starting ES (Figure 7d,f). Altogether, these preliminary results indicate that PCMFs-assemblies interconnected by 35N LT-microwires can be used for long-term ES in vivo.

## 4. Discussion

Biofunctionalized PCMFs are promising candidates for the fabrication of electroactive scaffolds able to promote neural regeneration [9,18,20,24]. PCMFs may be particularly useful for spinal cord repair because they can promote guided, long-distance axonal regrowth and cell migration, while still enabling the electrical stimulation of the regenerating tissue with high spatial resolution. However, the development of this technology has been hampered by the relatively low electrochemical stability of the microfibers [19] and the challenging problem of their interconnection for electrical supply in the living organism. Considering that human spinal cord lesions extend longitudinally for about 2 cm in average [21,22], the present study used PCMFs of mm to cm in length and investigated their electrical performance in vitro and in vivo. Current-controlled biphasic stimulation was used, applying electric intensities known to activate neurons and axonal tracts [19,26]. The results indicate that PCMFs of >6 mm in length can be used to fabricate implantable electroactive scaffolds able to stand neuro-regenerative, sub-chronic ES, and provide a baseline of knowledge regarding the PCMF performance that may guide further optimization steps towards their clinical application.

The PEDOT coating of electrodes, including carbon microfibers, substantially reduces the voltage produced for an applied electric current, thus preventing electrochemical reactions and enabling the safer electrical stimulation of biological tissues [17,18,19]. Nevertheless, this enhancement in electrode performance is temporary because the polymer deteriorates at variable speeds depending on its molecular composition, underlying electrode surface, environment of operation, and electrical parameters [19,32,33]. Controllable factors that may lead to polymer failure include the insufficient attachment of the polymer to the substrate [32,34], dopants exhibiting low oxidation potential or high mobility [34,35], excessive solvated ion transport in/out the polymer coating [4,36], and application of high electric charge densities [19], among others. In vivo conditions impose additional challenges to PEDOT and electrodes, particularly the inflammatory response, mechanical stress, limited diffusion of electrochemical species, and ionic concentrations, further limiting their electrical functionality [32].

We found a minimum length requirement of 6 mm for PCMFs to stand the 2 million pulse ES protocol (6.09 μC/cm^2^/phase) with minimum changes in voltage excursions in vitro. Nevertheless, the EIS and SEM analysis of 6 mm long PCMFs already showed some electrical and mechanical deterioration after pulsing. On the other hand, 1 mm PCMFs submitted to 800k pulses (36.53 μC/cm^2^/phase) showed a marked deterioration, and it is reasonable to expect a more severe degradation of the polymer and the carbon substrate if those microfibers had completed the 2 million pulse protocol. Thus, our cylindrical PCMFs exhibited lower stability when compared to the PEDOT-coated electrodes of gross two-dimensional shapes, and also allowed for a lower electric charge density application. For example, circular roughed platinum electrodes (200 μm diameter) coated with PEDOT:PSS, PEDOT:p-toluene sulfonic acid (PEDOT:pTS) or PEDOT:LiClO_4_ [34] tolerated up to 864 million pulses at 160 μC/cm^2^/phase before starting to deteriorate. The 80 μm diameter platinum electrodes coated with PEDOT:pTS also produced safe voltages for about 127 million biphasic current pulses at 400 μC/cm^2^/phase [33]. The higher stability of PEDOT in those studies may be attributable to the small dopants used, the enhanced surface roughness, the higher thickness of the PEDOT coating, and the metallic underlying electrode. The role played by the chemical composition of the substrate in the stability of composite electrodes is unclear; however, PEDOT-coated glassy carbon (GS) electrodes, more akin to PCMFs, apparently exhibit lower stability compared to PEDOT-coated metallic electrodes. For instance, the arrays of PEDOT:PSS-coated GC electrodes (300 μm in diameter each) could withstand 5 million biphasic current pulses (430 μC/cm^2^/phase) without changes in impedance and no signs of polymer coating cracking in SEM imaging [35]. However, when the GC-electrodes were miniaturized to 60 μm in diameter and submitted to the same electrical pulsing protocol (resulting in 10 mC/cm^2^/phase), they only tolerated 1 million pulses.

Considering the morphological changes detected by the SEM imaging of PCMFs, we postulate the irreversible mechanical disruption and the delamination of the polymer due to swelling by transport of hydrated ions and polymer overoxidation as the main factors leading to microfiber deterioration. Delamination is a common mode of failure in PEDOT:PSS films [34]. This polymer increases in mass simply by their immersion in electrolyte solutions due to the diffusion of solvated ions [36,37,38,39], and the swelling effect occurs faster if the electrolyte contains inorganic ions [36,37]. Due to the absence of macromolecules, such as proteins in the electrolyte solution (ACSF), we used PCMFs for testing in vitro, which may have further facilitated ion transport and contributed to disruption of the polymer coating. When polymer films are submitted to cyclic voltammetry (CV), the continuous mass exchange between the electrolyte and the polymer due to the ion drifting in and out with reduction and oxidation can cause large changes in the viscoelastic properties of polymer [38], thus limiting the device stability and reversibility. Actually, the PSS and PEDOT chains of the polymer are repeatedly repelled apart and then brought closer during each cycle of voltammetry, and polymer cracking and delamination is easily visible [32,34,39,40]. Once initiated, mechanical disruption might be potentiated by other phenomena such as polymer overoxidation with the cleavage of C-C bonds [41], producing further changes in the morphology and formation of crevices in the polymer film. 

Mechanical disruption of the conducting polymer due to swelling and overoxidation may be further facilitated by the long and cylindrical shape of PCMFs, and by the relatively low electrical conductivity of carbon microfibers (about 10^4^ S/m) when compared to metals (about 10^7^ S/m). These factors probably lead to heterogeneous electric charge distribution in the axial and transverse planes of the microfibers, thus creating voltage gradients and anisometric swelling by hydrated ion transport in the polymer coating. This may produce multifocal mechanical stress with lateral and longitudinal forces detaching the polymer coating from the underlying surface and producing radial and circumferential fractures in the polymer, as visualized by SEM. The same factors might cause the overoxidation of some polymer regions, further contributing to the delamination and appearance of fractures in the polymer shell, as well as the buckling of the whole structure, as predicted for composite conductive microwires [42,43].

EIS has been extensively used as a reliable technique for the study of conducting polymer coatings [4,17,18,27,32,34,35,44,45,46,47,48,49,50,51,52,53,54,55]. EIS provides useful data about electric charge transfer processes occurring across the substrate and the polymer, and helps elucidate the mechanisms involved in the loss of electrical performance. In fact, EIS plots showing a progressive increase in the impedance modulus directly correlate with the degradation or delamination of the conductive polymer coating [35]. Besides the impedance values obtained from EIS Bode and Nyquist plots, modelling the equivalent circuit that best fits EIS data provides relevant information regarding electronic parameters, ion transport, and electrode capacitance. Several equivalent circuits have been used to interpret EIS measurements from conducting polymers [4,17,18,27,32,34,35,44,45,46,47,48,49,50,51,52,53,54,55]. Some of those circuits [48,51] are complex and include the electrolyte and pore resistances, the capacitance of the organic coating, and a parallel association of the double layer capacitance and the polarization resistance, the latter representing the processes in the interphase between the organic coating and the metal substrate. In some cases [45,51], a Warburg impedance describes the diffusion processes into and out of the polymer coating. Novel interpretations for EIS data of PEDOT coatings have identified the transport mechanisms inside the polymer as an anomalous diffusion process defined by the parabolic subdiffusion equation [18,46], or the hyperbolic equation [55]. In the present work, we applied an electrical circuit with a few parameters to fit EIS data and characterize charge transfer and transport mechanisms inside the polymer. The circuit including generalized elements was based on published models [27] frequently used to interpret EIS data from conducting polymers [4,49,50,52,53]. The values obtained for electronic capacitance (Cd), diffusional pseudo-capacitance (C_D_), and diffusional resistance (R_D_) are particularly useful as they represent the main determinants of electrical working of the PEDOT coating.

Analyses of EIS data from PCMFs before starting ES showed that C_D_, and to a lesser extent C_d_, increased with microfiber length and polymerization charge, in agreement with studies of PEDOT coatings on other types of electrodes [27,49,50]. C_d_ was about an order of magnitude higher than C_D_ in our small PCMFs and hence, the latter became the limiting factor for electric transfer through the polymer bulk and conditioned the total capacitance of the microfibers. An inverse proportion between C_d_ and C_D_ was reported for larger electrodes [27,49,50]. Nevertheless, some authors [52] found values for C_d_ in the same order of magnitude as C_D_, whereas others, using microelectrodes with a gross surface area smaller than PCMFs [53], obtained data in line with our results. Calculated C_d_ and C_D_ values indicate that the polymer became more restrictive to ion transport with the reduction in the microfiber surface area. Although possible mechanisms need further investigation, it is likely that the low electric charge needed for electropolymerization limited to some extent the incorporation of the large dopant molecules (PSS-*co*-MA). The same phenomenon might increase R_D_ in the shortest PCMFs. EIS circuit modelling evidenced that the R_D_ of 1 mm PCMFs doubled or tripled the R_D_ value of longer microfibers before ES. Previous studies [27,49] found this magnitude to be independent of coating thickness, whereas others [18,53] observed a slight increase in R_D_ with polymerization charge. Moreover, R_D_ was inversely proportional to electrolyte concentration [27], or varied with the ions in the testing solution [49]. Our results suggest that R_D_ also depends on the electrode area and therefore on the electric charge used for the polymerization.

C_d_, C_D_, and R_D_ were also useful to follow polymer changes after repetitive ES. In particular, C_D_ seemed to be the most reliable parameter to follow polymer deterioration. For 6 mm long PCMFs, about 34% of C_D_ was lost after 2 million ES pulses, in agreement with the morphological changes without the gross detachment of the polymer, and despite the lack of significant increase in voltage excursions during pulsing. This suggests that EIS is more sensitive than voltage excursion measurements with 200 μs ES pulses for assessing the performance of organic electrodes, likely because EIS spectra include low-frequency sinusoidal waves allowing for the better detection of electric transfer associated with slow ion transport processes; whereas electric transfer in short ES pulses is more dependent on electronic parameters. In fact, contrary to diffusional pseudo-capacitance, the electronic capacitance (C_d_) of PCMFs was enhanced by more than 300% after 2 million ES pulses, indicating a positive conditioning effect of repetitive electrostimulation on the polymer. Although R_D_ increased by 22% after 2 million ES pulses, its behavior was less consistent and could not be easily ascribed to the electrical performance of the polymer. 

The circuit we used for modeling EIS data accounts for electric charge transport through the conducting polymer and the electrolyte, without considering the carbon microfiber itself. Because PCMFs deteriorate and the polymer detaches after ES, exposing the carbon surface, other mathematical models might provide additional information on the electrical transfer and loss of electrical performance. Adding the charge transfer resistance (R_ct_) and the double-layer capacitance (C_dl_) of the electrode surface in parallel to the circuit we used allowed other researchers [49,54] to characterize processes taking place between the coating and the substrate. This addition to the model can be generalized using CPEs instead of ideal capacitors to represent the double layer and electronic storage effects [56]. R_ct_ and C_dl_ were associated with the appearance of a semicircle at the highest frequencies in the Nyquist plot [54], or a depressed semicircle when the double layer was described by means of a CPE [57]. In our measurements, semicircles were not clearly identified at frequencies > 1 kHz, thus precluding the study of electrical processes at the carbon microfiber/polymer interphase. Irrespective of this, C_dl_ has a very low magnitude compared with either C_d_ or C_D,_ and therefore, its values are prone to calculation errors in the presence of polymer coating. Nevertheless, the exposition of the carbon microfiber surface to the electrolyte through the polymer cracks should produce resistive components spatially separated along the electrode surface, acting heterogeneously in the EIS spectra. Further improvement in the measurement settings will allow us to include additional circuit elements for a more complete follow-up of the evolution of polymer degradation and cracking, providing valuable information for PCMF optimization.

The fabrication of mechanically and electrically stable, implantable PCMF-scaffolds is necessary for advanced applications of this technology in neural repair. With regard to their potential harmfulness, carbon fibers are known for being a relatively chemically inert material [31]. Intratracheally or intraperitoneally injections of CMFs produce no acute toxicity in mice, evaluated as lung histopathology and gene expression [58]. In contrast, other carbon-based materials like carbon nanotubes cause cell membrane damage and genotoxicity when added to human lung epithelial cells in vitro [59]. The good biological compatibility of CMFs coated with conducting polymer (i.e., PCMFs) and further functionalized with biomolecule multilayers was initially addressed in vitro, where they induced the axonal growth and excellent survival, proliferation, and migration of glial cells [23]. Furthermore, when implanted into the uninjured rat spinal cord in vivo, functionalized PCMFs evoked no persistent fibrosis or inflammation, establishing direct contact with neuronal bodies, axons, and dendrites in the long term [20]. However, achieving appropriate electrical interconnection of carbon materials for use in vivo is difficult because of the fragility of carbon microstructures and because the interconnections may trigger further inflammation and fibrosis at neural injury sites. Large interconnections may directly impede tissue regrowth, which is in fact the reason for implanting the scaffolds. Moreover, handling individual microfibers and keeping their alignment within a large lesion is an extremely laborious task. To be clinically useful, the microfibers must be conveniently presented as an assembly within a gel suitable for implantation. Previous studies with PCMFs have succeeded in their implantation as bundles within alginate or fibrin gels [20,24]. Although some neuro-reparative responses were observed, those bundles were less controllable in terms of microfiber spacing and alignment and had no interconnection for ES. Here, we advanced in the production of an interconnected PCMFs assembly with defined geometrical features, and demonstrated the feasibility of implanting the assembly for application of ES within the injured spinal cord. Medical grade electroconductive epoxy produced durable electrical contacts between the metallic microwires and PCMFs, able to stand long-term electrical stimulation in vivo. To the best of our knowledge, no previous examples exist of carbon-based electroconducting scaffolds implanted in the central nervous system with an interconnection for electric supply. Uncoated carbon microfibers were used for neural recordings in the rodent brain, but the large interconnection for electric supply remained outside the head of the animal and only the microfiber tips entered the neural tissue [60,61,62]. Polypyrrole/silk fibroin conduits, applying a small voltage gradient through wires secured to the conduit ends, were used to repair rodent peripheral nerves [63]. However, no assessment of the electric current transfer through the implanted composite conduits was performed in vivo, and therefore, the suitability of those devices for neural ES is uncertain. Similar approaches used scaffolds containing carbon nanotubes (CNTs) to bridge sciatic nerve lesions [64,65]; however, in these cases, there was no electrical interconnection, and ES was applied by electrodes on nerve segments distant from the implants. Hence, our results provide a baseline of technology that may be further improved to achieve electrically conducting tissue scaffolds. Improvements aimed at preventing polymer disruption and detachment from the CMF in the course of chronic ES may include chemical engineering, such as covalent bonding of PEDOT to the CMF surface, or the introduction of small dopants that could confer stability to the polymer while avoiding excessive accumulation of solvated ions. Eventually, designing novel conducting polymers with enhanced physical and mechanical properties like flexibility and elasticity [66] would further improve the ability of the scaffold to support neural regeneration and electrical interfacing in vivo.

## 5. Conclusions

This study demonstrates that the PCMFs of mm to cm in length, either as individual microfibers or as implantable assemblies, can be used for the application of repetitive biphasic electric current pulses of duration and intensity relevant to reparative neurology. The chronopotentiometry and EIS analyses of individual PCMFs during stimulation with biphasic pulses revealed that microfibers of 6 mm or longer may enable sub-chronic ES under electrochemically safe voltages. However, equivalent electrical circuit modeling from impedance data still detected a slight electrical deterioration of the PCMFs, likely caused by the structural damage of the polymer coating as visualized by SEM. On the other hand, interconnected PCMF-assemblies could be implanted in a model of porcine spinal cord injury and allowed safe, prolonged neural stimulation in vivo. The data provided in this work regarding PCMFs deterioration and its possible mechanisms may guide the assessment of microfibers stability and the design of optimization strategies to achieve mechanically and electrically stable active tissue scaffolds for human application. 

## 6. Patents

J.E. Collazos-Castro and G.R. Hernández Labrado are inventors in patent ESP201231969 regarding PCMFs; and J.E. Collazos-Castro together with A. Alves-Sampaio are co-inventors in patent application P202230626 on fibrin/PCMFs scaffolds.

## Figures and Tables

**Table 1:** Electrical parameters for PEDOT:PSS-*co*-MA polymerization depending on microfiber length.

CMF Length (mm)	CMF GSA (μm^2^)	Polymerization Current (nA)	Polymerization Charge (μC)
1	22,018	22.01	42.27
2	43,998	43.99	84.47
4	87,958	87.95	168.88
6	131,918	131.91	253.28

**Table 2:** EIS data fitting for PCMFs of different lengths. R_s_ represents the solution resistance, and the other parameters define CPE and Z_GD_ elements according to Equations (1) and (2). C_tot_ is obtained from the series association of the capacitances C_d_ and C_D_.

CMF Length	R_s_/kΩ	C_d_/μF	R_D_/kΩ	τ_D_/ms	C_D_/μF	C_tot_/μF
1 mm	15.75	3.98	10.70	14.61	1.37	1.02
2 mm	15.35	20.42	3.37	8.15	2.42	2.16
4 mm	18.35	33.81	3.15	19.95	6.34	5.34
6 mm	12.84	75.00	5.01	34.47	6.88	6.30

**Table 3:** Electrical values for 6 mm length PCMFs at different periods of ES, obtained from EIS data fitting.

Pulses	R_s_/kΩ	C_d_/μF	R_D_/kΩ	τ_D_/ms	C_D_/μF	C_tot_/μF
0k	12.84	75.00	5.01	34.47	6.88	6.30
40k	13.54	27.88	3.89	23.37	6.00	4.94
400k	11.54	251.57	2.55	11.66	4.58	4.50
800k	13.65	433.66	2.91	14.22	4.88	4.82
1200k	13.20	519.08	4.19	18.46	4.40	4.36
1600k	14.31	231.45	5.50	22.18	4.04	3.97
2000k	14.77	232.65	6.47	29.55	4.57	4.48

## Data Availability

Data are contained into the article. Further inquiries can be directed to the corresponding author.
